# Molecular mechanism of atrial remodeling in patients with aging atrial fibrillation under the expression of microRNA-1 and microRNA-21

**DOI:** 10.1080/21655979.2021.2008668

**Published:** 2021-12-25

**Authors:** Kexin Yuan, Pei Zhao, Lili Wang

**Affiliations:** a Department of Cardiovascular, Hebei People’s Hospital, Shijiazhuang, China; b Department of Laboratory Medicine, Hebei People’s Hospital, Shijiazhuang, China

**Keywords:** microRNA-1, microRNA-21, aging atrial fibrillation, atrial remodeling, cell apoptosis

## Abstract

We investigated the expression levels of microRNA-1 (miRNA-1) and microRNA-21 (miRNA-21) in the atrial tissues of patients with atrial fibrillation (AF) and the molecular mechanism of action in atrial remodeling. Patients with valvular heart disease were selected as the subjects. The ultrastructure, degree of myocardial fibrosis, apoptosis index (AI), expression of microRNA-1, expression of microRNA-21, and mRNA of TIMP-1, MMP-9, BCL-2, and Bax of patients were compared and analyzed in each group. The results showed that the degree of myocardial fibrosis and AI in patients with AF of the same age were extremely higher than those of patients with sinus rhythm (SR) (*P* < 0.01). Patients with AF showed much higher messenger RNA (mRNA) levels of mini-mental Parkinson 9 (MMP9) and Bax and obvious lover mRNA levels of tissue inhibitors of metalloproteinase 1 (TIMP-1) and Bcl-2 compared with patients with sinus rhythm (SR) (*P* < 0.05). It indicated that the expression of miRNA-1 in the AF patients was markedly down-regulated, and that miRNA-21 was up-regulated. This showed that microRNA-1 and microRNA-21 were involved in the molecular remodeling of aging AF through the regulation of primers, which would provide a critical basis for diagnosis and treatment of aging AF.

## Graphical abstract

**Figure:**
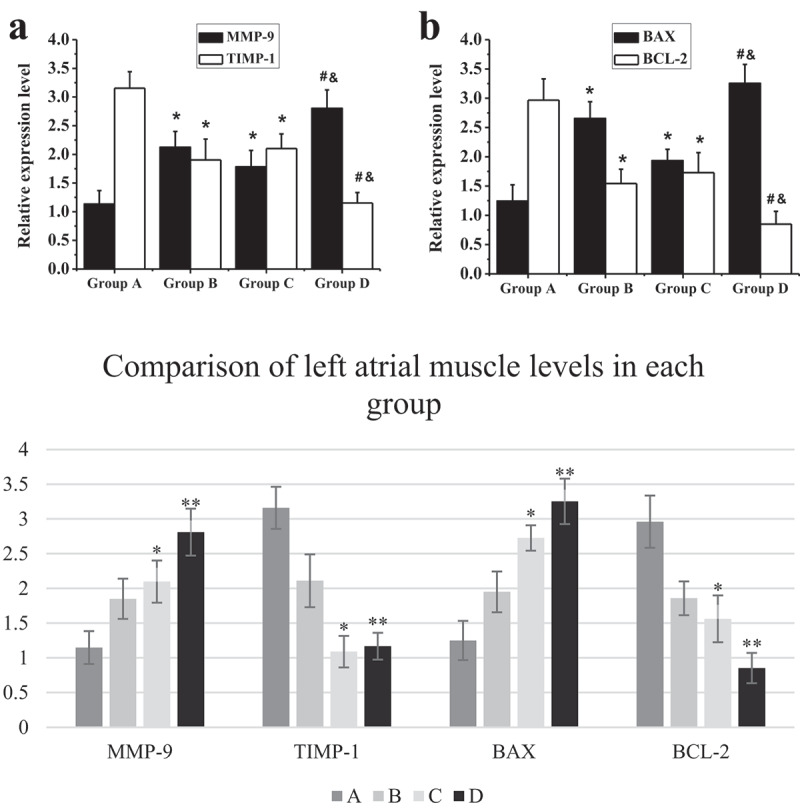

## 1. Introduction

Atrial fibrillation (AF) is a common persistent arrhythmia disease [1]. It is more common in the elderly, and its prevalence increases with age [2]. Relevant data show that the prevalence of AF is only about 1–2% in the general population but higher than 15% in the elderly over 80 years old [3]. In addition, relevant epidemiological statistics indicate that the average age of patients with AF is 75 years old, and about 70% of the patients are 65–85 years old [4]. AF can increase the risk of stroke and heart failure, and is closely related to the emerging of cardiovascular disease, hypertension, coronary artery disease, and diabetes. In addition, aging greatly increases the emerging of atrial arrhythmias, especially AF [5]. Aging can cause changes in the structure of the atria and pulmonary veins and electrophysiological remodeling, so it is an independent risk factor of AF [6]. In view of the research on the relationship between aging and AF, some scholars have put forward the concept of aging AF, and actively exploring the pathogenesis and treatment of aging AF has become a hot research topic in cardiovascular diseases.

As a non-coding ribonucleic acid (RNA), micro-ribonucleic acid (miRNA) mainly regulates gene expression after transcription [7]. Under normal circumstances, miRNAs present high expressions in the cardiovascular system, while they show a decreased level in the lesion of vascular disease. Under the condition of age-related AF, many myocardial cells will show apoptosis and loss, which in turn leads to hypofunction of the heart and the atrial remodeling [8]. Studies have shown that miRNA plays an important role in myocardial remodeling caused by various cardiovascular diseases [9,10]. Moreover, miRNA-21 has great effects on the post-transcriptional regulation of myocardial fibrosis-related genes during myocardial remodeling [11]. If AF occurs, anti-apoptotic and pro-apoptotic proteins can coordinately regulate various stress responses, and miRNAs also participate in the regulation process of programmed cell death after gene transcription [12]. The miRNA-1 plays a key role in the apoptosis mediated by oxidative stress in myocardial cells and is closely correlated to the emerging and development of AF [13]. There is little literature on the regulatory mechanism of miRNAs related to aging AF remodeling, atrial fibrosis, and apoptosis, and there are few reports on the influence of miRNA-1 and miRNA-21 expression on aging AF.

Therefore, this study was to explore the molecular mechanism of atrial remodeling in patients with aging AF based on the expression levels of microRNA-1 and microRNA-21. The right atrial appendage tissue, ultrastructure, degree of myocardial fibrosis, and apoptosis index (AI) of atrial tissue cells of patients were recorded and compared in each group. In addition, the expression of microRNA-1 (miRNA-1), microRNA-21 (miRNA-21), and tissue inhibitors of metalloproteinase 1 (TIMP-1), mini-mental Parkinson 9 (MMP9), Bcl-2, and Bax mRNA levels were determined by reverse transcription-polymerase chain reaction (RT-PCR), aiming to provide a theoretical basis for the pathogenesis and clinical treatment of AAF.

## 2. Materials and methods

### 2.1. Subjects investigated and grouping

One hundred and twenty-six patients with valvular heart disease, who underwent cardiac surgery in the Hebei People’s Hospital from October 2017 to October 2018, were selected as the subjects investigated, including 74 males and 52 females, with the average age of 54.65 ± 34.23 years old. There were 80 patients with sinus rhythm (SR) and 46 patients with persistent AF. According to whether the subjects were over 65 years old and whether they suffered from persistent AF, all the patients were divided into 4 groups (group A: non-elderly patients with SR (younger than 65 years old, 36 cases), group B: non-elderly patients with AF (younger than 65 years old, 18 cases), group C: elderly patients with SR (older than 65 years old, 38 cases), and group D: elderly patients with AF (older than 65 years old, 34 cases)). The experiment had been approved by the Ethics Committee of Hebei People’s Hospital, and all the patients included in the study had signed informed consents.

The criteria for inclusion were defined to include patients with SR who were over or equaled to 65 years old; patients with SR or persistent AF and over 65 years old; patients who underwent cardiac surgery; and patients who were preoperatively diagnosed by echocardiography, electrocardiogram, and 24-h dynamic electrocardiogram.

The criteria for exclusion were defined to include patients suffering from diabetes, hypertension, and hyperthyroidism disease; and patients who had damage, infection, and electrolyte disorders of liver and renal function as shown in Table 2.

**Table 1.** The primer sequences

Gene name	Sequence	Length (bp)
GAPDH 87	5ʹ- TGCACCACCAACTGCTTAGC-3ʹ 5ʹ- GGCATGGACTGTGGTCATGAG-3’	87
HCN2 130	5ʹ- CCAGCTGTAAGACAGGGACG-3ʹ 5ʹ- GCGGGCCAAGTAITGCACIT-3’	130
HCN4	5ʹ- GGGGAATTCGCA ACTGA AGC-3ʹ 5ʹ- TGCTGCGCCCTTAAATCTCT-3ʹ 5ʹ- AGTGCGTGTCGTGGAGTC-3’	168

**Table 2.** Comparison of the basic data of the patients

	Group A	Group B	Group C	Group D	*P*
Number of the patients	36	18	38	34	
Gender (male/female)	23/13	10/8	23/15	23/11	>0.05
Age (years old)	40.26 ± 9.34	43.13 ± 9.35	69.34 ± 4.28	68.98 ± 10.12	>0.05
Ejection fraction (%)	42.83 ± 4.38	42.28 ± 6.28	42.35 ± 8.36	41.25 ± 8.35	>0.05
Mitral valve replacement	13	10	15	13	>0.05
Mitral valve + aortic valve replacement	7	6	9	5	>0.05

### 2.2. Collection of the atrial muscle specimen

All the patients were generally anesthetized and underwent valve replacement surgery under extracorporeal circulation. 200 mg of the right auricular tissues were quickly taken before the extracorporeal circulation and were rinsed with normal saline, and their fat was removed. The part of the obtained atrial tissues was placed in liquid nitrogen to store for later use, and the rest of the tissues were fixed with 4% paraformaldehyde solution. The above operations were completed quickly within 1 minute. For the patient who underwent extracorporeal circulation for valve replacement surgery, the conventional treatment included the right auricular cannula. A small piece of the auricular tissues before the cannula would not cause any adverse effects the valve replacement surgery and the prognosis of patients.

### 2.3. Ultrastructure detection of myocardial tissues of the right atrium

The myocardial tissues of the right atrium were cut into0.5 cm^3^ sized tissue blocks, which were rinsed with 0.85% normal saline to remove blood stains and quickly fixed with 4% paraformaldehyde solution. After that, they were rinsed with running water for 24 hours. Then, the gradient concentration alcohol in sequence was used for dehydrating the blocks for 10 hours with 30%, 50%, and 70% alcohols, for 3 hours with 80%, 90%, and 95% alcohols, and for 15 minutes with 100% alcohol I and II. After that, the blocks were soaked with the mixed solution of 1:1 ratio of xylene and anhydrous alcohol for 2 hours, and then placed in pure xylene for 20 minutes and set in a thermostat that was higher than the melting point of paraffin wax for infiltration. The tissue blocks were marked and placed in wax liquid for embedding, made into thin slices with a thickness of about 50–100 nm, and then stained sequentially with lead citrate and uranyl acetate to observe the slices under an electron microscope after drying.

### 2.4. Masson staining

The myocardial tissues of the right atrium fixed by 4% paraformaldehyde solution were dehydrated, immersed in paraffin, cleared, and embedded. The slicer was employed to prepare the section with a thickness of 5 μm. The section was for routine deparaffinization, stained with Harris hematoxylin for 3 minutes, and rinsed with running water. It was differentiated with 1% hydrochloric acid alcohol for 3–5 seconds. The differentiated section was rinsed with running water. It was returned to blue by warm water for 1 minute and then washed with running water; and it was stained with ponceau-acid fuchsin for 3 minutes and rinsed with distilled water. After that, the section was differentiated with 1% phosphomolybdic acid for 1 minute. After the remaining liquid was wiped off, the section was re-stained with 2% aniline blue for 1 minute, and then rinsed with 95% alcohol. Next, it was dehydrated with 95% alcohol and absolute alcohol and sealed with neutral gum after the tissue section was air-dried. The tissue section was observed under an optical microscope, and 5 fields of view were selected for taking pictures randomly, and the myocardial collagen volume fraction (Collagen Volume Fraction, CVF) was measured by Image-Pro Plus 6.0 image analysis system [14].

### 2.5. Detection technology of terminal deoxynucleoitidyl transferase-mediated nick-end labeling

The paraffin sections were soaked and rinsed with xylene, 100% ethanol, 95% ethanol, 75% ethanol, and polybutylene succinate phosphate buffer solution (PBS) in sequence, added with Proteinase K solution (20 μg/mL) to completely cover, incubated at room temperature, and rinsed with PBS for 2–3 times. PBS containing 2% H_2_O_2_ was added into the color cylinder to react for 5 minutes, and then the mixture was rinsed with PBS 2–3 times. The terminal deoxynucleoitidyl transferase terminal transferase (TdT) enzyme buffer was added to keep the cells in a moist state, and they were incubated at 37°C for 1 hour. After that, they were placed in the staining jar, and the 37°C buffer was employed to stop the reaction. Next, the sections were for heat preservation for 30 minutes, washed with PBS solution three times with 5 minutes each time. Anti-digoxigenin antibody was added dropwise into them to react for 30 minutes at room temperature and cleaned with PBS. 0.05% diaminobenzidine (DAB) was added dropwise into them, the mixture was left for 5 minutes, and then, distilled water was used for rinsing them three times for 1 minute each. The sections were stained with methyl green solution for 10 minutes, washed with distilled water and n-butanol in sequence, and dehydrated with xylene three times. After sealing and drying, the sections were observed under an optical microscope and the apoptosis index (AI) was calculated [15].

### 2.6. Detection by reverse transcription-polymerase chain reaction

The total RNA and total miRNA in the myocardial tissues of each group were extracted to detect the absorbance A at 260 nm and 280 nm of the total RNA, respectively; and the total RNA was quantified based on the ratio of A260/A280. Afterward, the above-mentioned total RNA template was taken 2 μL, and the reverse transcription kit All-in-One^TM^ miRNA qRT-PCR was employed to synthesize the first strand of complementary DNA (cDNA). The cDNA was used as a template and added with 2× All-in-One qPCR Mix, specific primers, and water for amplification. What is more, hsnRNA U6 was regarded as the internal reference primer. The comparative CT method was used for relative quantification, and the Pfaffl method was applied to calculate the relative expression. All the primers were designed by Primer designing tool software and synthesized by Sangon Biotech (Shanghai) Co., Ltd.

The primer sequence is shown in Table 1:

### 2.7. Extraction of total miRNA from myocardium

First, 1 mL of TRIZOL reagent was added to the 1.5 mL Eppendorf (EP) tube, which was placed on ice for pre-cooling. Secondly, the frozen myocardial tissue was taken from the cryotube, and about 100 mg of myocardial tissue was taken and wrapped in aluminum foil. During this period, the aluminum foil wrapped with myocardial tissue was repeatedly put in liquid nitrogen to ensure low temperature. After grinding into powder, the tissues were quickly transferred to the EP tube, shaken, and mixed. Thirdly, the tissues were centrifuged at 12,000 g for 5 minutes at 4°C to discard the precipitate, and then the supernatant was transferred to a new EP tube. Fourthly, 200 uL of chloroform was added, shaken, mixed, and placed at room temperature for 2 minutes. Fifthly, the solution was centrifuged at 12,000 g for 15 minutes at 4°C, the upper aqueous phase was carefully sucked and transferred into a new EP tube. Sixthly, 500 uL of isopropanol was taken and mixed upside down, placed at room temperature for 10 minutes, and centrifuged at 12,000 g at 4°C for 10 minutes. At this time, a white precipitate was visible, which was RNA. Seventhly, the supernatant was discarded, the residential substance was rinsed with 75% ethanol and centrifuged at 8000 g for 5 minutes at 4°C. Next, the clear liquid was discarded, and the deposit was dried at room temperature. Then, RNA was dissolved in 50 μL denuclease (DEPC treatment) water and frozen at 80°C for later use. Finally, a sample of total RNA was taken to measure the optical density at 260 nm and 280 nm with an ultraviolet (UV) spectrophotometer, and estimate the purity of RNA by calculating the ratio of the UV absorbance at 260 nm and 280 nm (A260 nm/A280 nm) [16].

### 2.8. Statistical analysis

The experimental data were analyzed by SPSS22.0 statistical software, the measurement data were expressed as mean ± standard deviation, and the t test was employed to compare the differences among the groups. The count data were represented as percentages, and the χ^2^ test was applied to compare the differences among the groups. Moreover, the one-way factor analysis of variance (ANOVA) and least significant difference (LSD) method were used for the comparison among groups. If *P* < 0.05, the difference was statistically significant.

## 3. Results

### 3.1. Comparison on the basic data of the patients in each group

A total of 126 subjects investigated were selected in the study, including 36 non-elderly patients with SR, 18 non-elderly patients with AF, 38 elderly patients with SR, and 34 elderly patients with AF. The basic data of the patients are shown in Table 1. According to the data in Table 1, there was no statistical significance in the gender, age, ejection fraction, mitral valve replacement, and mitral valve + aortic valve replacement of patients from groups A and B and groups C and D (*P* > 0.05), so the data were comparable.

### 3.2. Morphological changes of myocardial tissues of the right atrium

**Figure 1.**
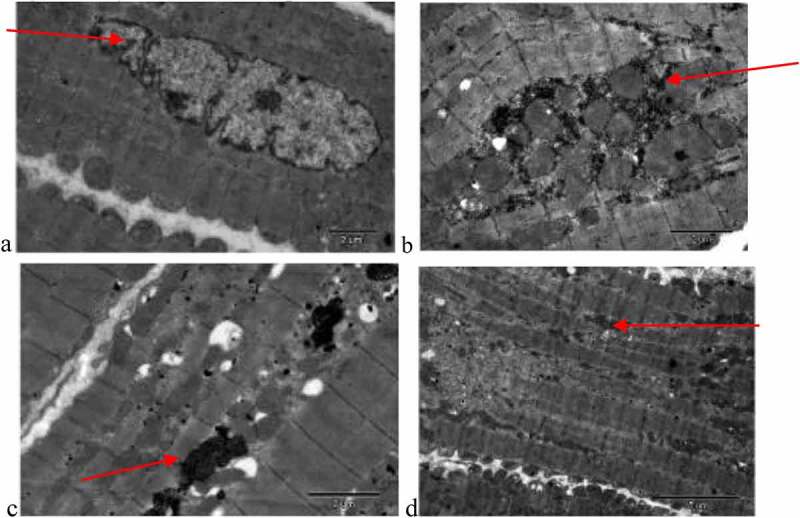
Ultrastructure of myocardial tissue cells (×10,000)

The ultrastructure images of the right atrium myocardial tissue cells in each group are shown in Figure 1. In Figure 1(a), the atrial cells of the patient in group A are regularly arranged with normal chromatin structure and uniform mitochondria. There was more serious degeneration of sarcomere, nuclear pyknosis, apoptosis, a small amount of secondary lysosomes, obvious mitochondrial swelling, endoplasmic reticulum swelling, less glycogen, and disordered muscle fibers in the patient of group B, as shown in Figure 1(b). Figure 1(c) shows that the sarcomere degenerated, the chromatin nucleus was pyknotic, the mitochondrial cristae swelled, the endoplasmic reticulum swelled, the glycogen increased, and muscle fibers became tight in the patient of group C. Figure 1 illustrates that the patients of group D showed the extremely serious degeneration of sarcomere, nuclear pyknosis, more apoptosis, more secondary lysosomes, vacuolar degeneration of mitochondria, obvious endoplasmic reticulum swelling, and myofilament collapse.

(a–d) were the images of the ultrastructure of myocardial tissue cells from groups A, B, C, and D, respectively.)

**Figure 2.**
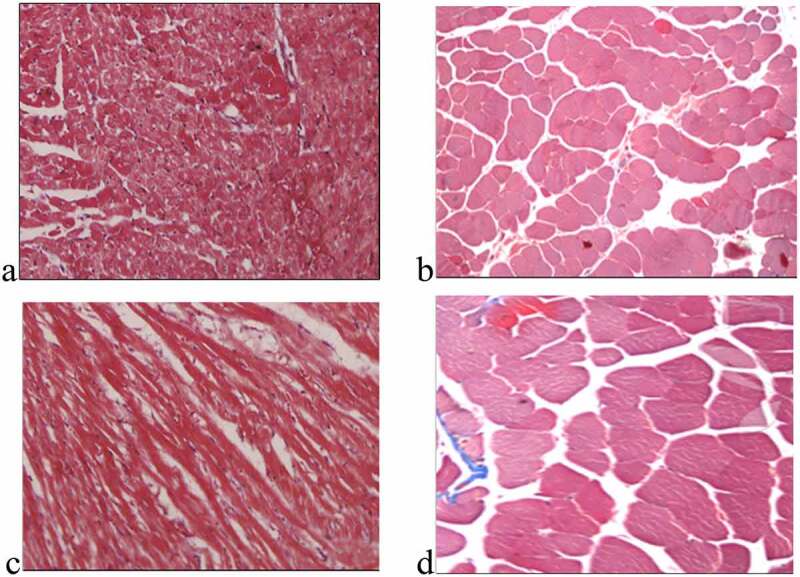
Fibrosis characteristics of the myocardial tissues (×160)

**Figure 3.**
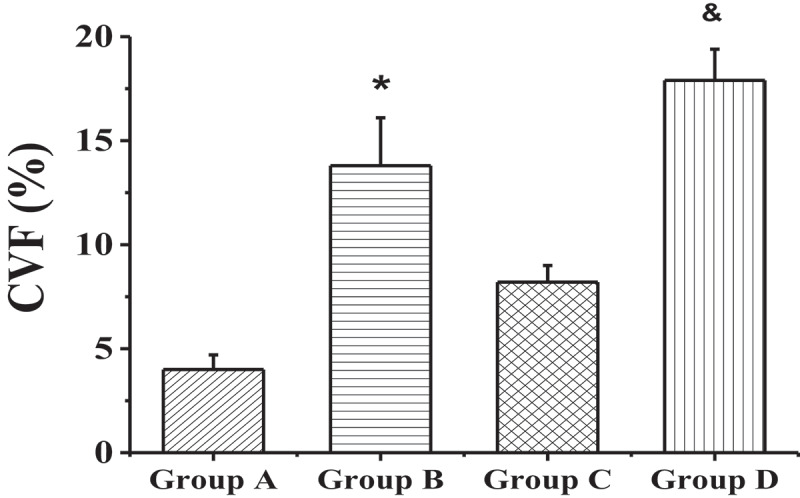
Comparison on the CVF values of the 4 groups

Figure 2 shows the staining results of myocardial collagen fibers in each group. Compared with the non-elderly patients, the myocardial tissues of the right atrium in the elderly patients had the denser and more compact fibrous tissue among the muscle cells, and a large amount of connective tissue separated the muscle bundles, showing excessive accumulation of collagen. The values of CVFs in groups A, B, C, and D were 4.0 ± 0.7%, 13.8 ± 2.3%, 8.2 ± 0.8%, and 17.9 ± 1.5%, respectively. The patients with AF of the same age had a higher degree of myocardial fibrosis than the patients with SR (*P* < 0.01), as shown in Figure 3.

(a–d) were the images of fibrosis characteristics of the myocardial tissues from groups A, B, C, and D, respectively.)

(* meant *P* < 0.01 compared with group A; and & represented *P* < 0.01 in contrast to group C.)

### 3.3. Results of terminal deoxynucleoitidyl transferase-mediated nick-end labeling staining

**Figure 4.**
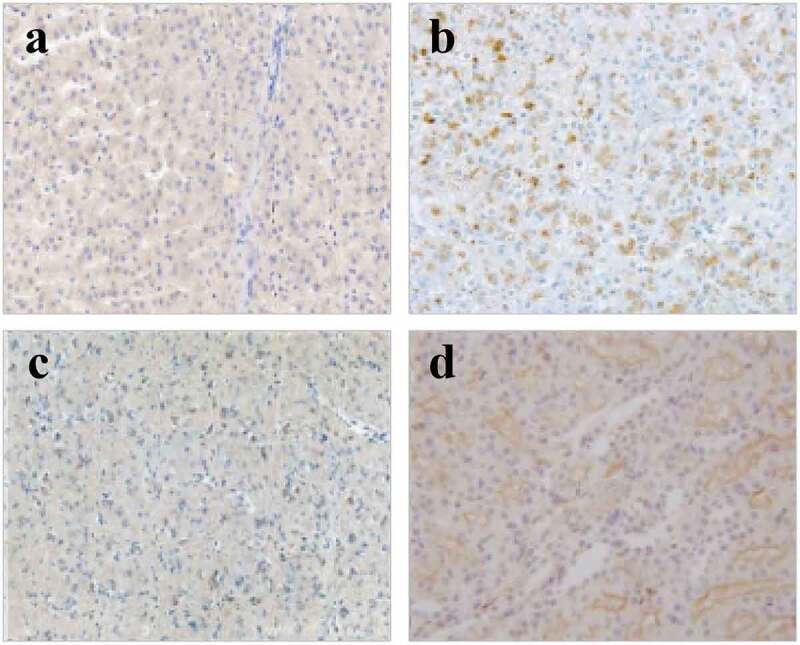
Results of myocardial tissues with TUNEL staining in each group (×400)

**Figure 5.**
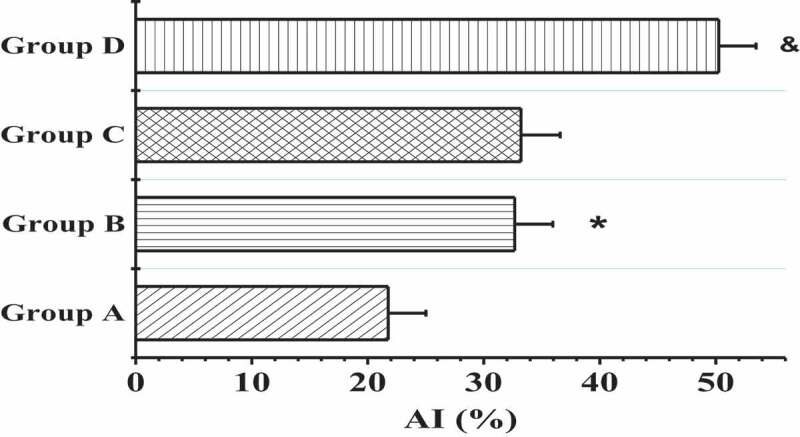
Comparison on the AI values of the 4 groups

The TdT-mediated dUTP nick-end labeling (TUNEL) was applied to detect the apoptosis of myocardial tissues in the right atrium of patients from four groups, and the results are shown in Figure 4 and Figure 5. In contrast to the patients in groups A and B, the patients in groups C and D showed more serious muscle cytolysis of the right atriums and higher TUNEL positive rates. When the nuclei were stained lightly with TUNEL, the heterochromatin had a more uniform distribution with an increase in volume (in Figure 4(a) and 4(b)). The AI values in groups A, B, C, and D were 21.76 ± 3.26%, 32.67 ± 3.29%, 33.21 ± 3.38%, and 50.25 ± 3.22%, respectively. In addition, the apoptosis degree of myocardial cells in patients with AF of the same age was markedly higher than that of patients with SR (*P* < 0.01); the positive rate of apoptosis in the elderly patients with AF was the highest; and the nuclei were pyknotic and had strong TUNEL staining, which was mainly characterized by extensive DNA lysis (Figure 4(c) and 4(d)).

(a-d) were the images of the TUNEL staining results of the patients from groups A, B, C, and D, respectively.)

(* meant *P* < 0.01 compared to group A; and & indicated *P* < 0.01 in contrast to group C.)

### 3.4. The messenger RNA expression levels of tissue inhibitor of metalloproteinase-1, matrix metalloproteinase-9, B-cell lymphoma-2, and B-cell lymphoma-2 associated X

**Figure 6.**
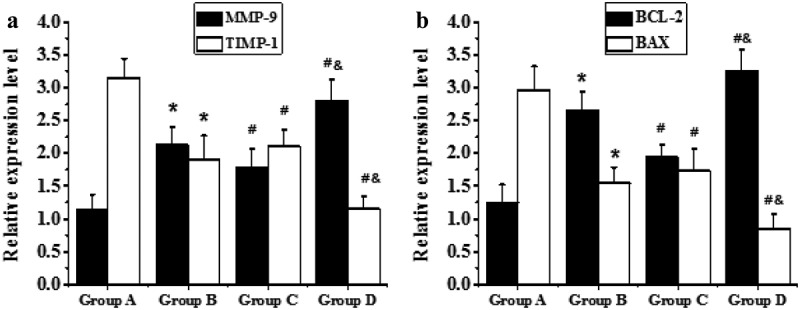
Comparison on the mRNA levels of TIMP-1, MMP-9, Bcl-2, and Bax

RT-PCR technology was adopted to detect the mRNA expression levels of TIMP-1, MMP-9, Bcl-2, and Bax in the myocardial tissues of the right atrium from all the patients in each group, and the results were demonstrated in Figure 6. The mRNA levels of MMP-9 and Bax in the elderly patients from groups C and D were substantially higher than those of the non-elderly patients from groups A and B, respectively (*P* < 0.05). The mRNA levels of MMP-9 and Bax in the patients with AF of the same age from groups B and D were remarkably higher than those of the patients with SR from groups A and C, respectively (*P* < 0.05). On the other hand, the mRNA levels of TIMP-1 and Bcl-2 in the elderly patients from groups C and D decreased sharply in contrast to the non-elderly patients from groups A and B, respectively (*P* < 0.05); and the mRNA levels of TIMP-1 and Bcl-2 in patients with AF of the same age from groups B and D reduced markedly in contrast to patients with SR from groups A and C, respectively (*P* < 0.05).

((a and b) showed the comparison on mRNA expression levels of MMP-9 and TIMP-1a nd mRNA expression levels of BAX and Bcl-2, respectively. * meant *P* < 0.05 compared with group A; # showed *P* < 0.05 compared to group B; and & indicated *P* < 0.05 in contrast to group C.)

**Figure 7.**
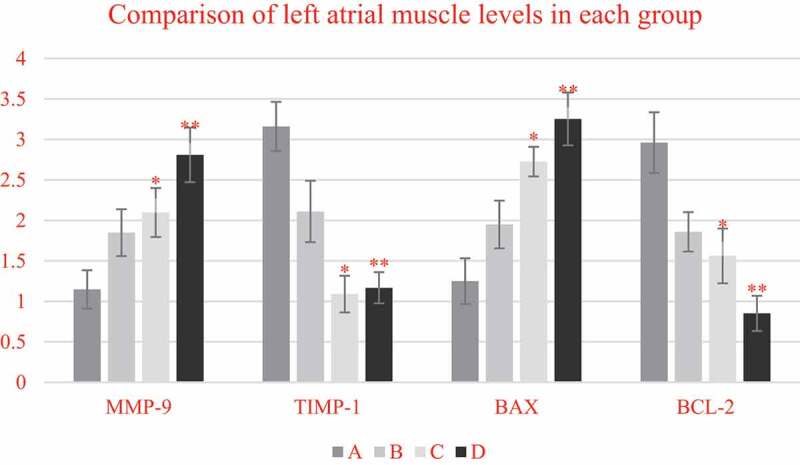
Comparison on mRNA expression levels of MMP-9/TIMP-1 and BCL-2/BAX in left atrium myocardial tissues of non-elderly patients and elderly patients with SR and persistent AF

Note: * suggested *P* < 0.05 compared with non-elderly patients with SR; and ** suggested *P* < 0.05 on comparison of patients with SR and AF in the same age group.

As shown in Figure 7, compared with non-elderly patients, the mRNA expression levels of MMP-9 and Bax in the left atrial muscle of elderly patients were significantly increased (*P* < 0.05), while the mRNA expression levels of TIMP-1 and Bcl-2 were significantly decreased (*P* < 0.05). In addition, a comparison between the patients with SR and patients with AF of the same age showed that the mRNA expression levels of MMP-9 and Bax in the two age groups of patients with AF showed significant upward-regulation trends (*P* < 0.05), especially in elderly patients with AF, so this upward trend was the most obvious (*P* < 0.05). On the contrary, TIMP-1 and Bcl-2 in the two age groups of patients with AF showed obvious downward trend in mRNA expression (*P* < 0.05), and such downward trend was the most obvious in elderly patients with AF (*P* < 0.05).

### 3.5. The expressions of microRNA-1 andmicroRNA-21 in myocardial tissues of the right atrium

**Figure 8.**
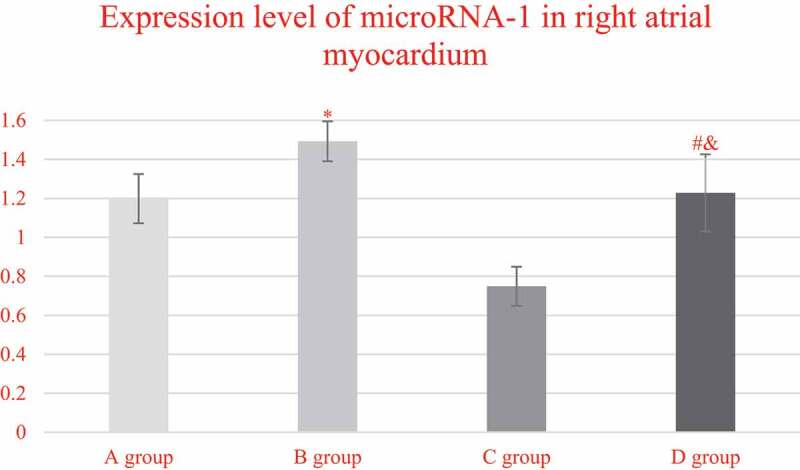
Comparison on the expression levels of miRNA-1 in the right atrium myocardial tissues of the patients from group A, C, and D

The expression levels of miRNA-1 in the myocardial tissues of the right atrium from each group, and the comparison results were illustrated in Figure 8. It was found that the expression levels of miRNA-1 in the right atrium myocardial tissues from the patients in groups A, B, C, and D were 1.1982 ± 0.1265, 1.4923 ± 0.1023, 0.7487 ± 0.1002, and 1.2983 ± 0.1976, respectively. In addition, the expression level of miRNA-1 in group C was steeply lower than that of group A (*P* < 0.05), while the expression in group D was obviously higher than the expression of group C (*P* < 0.05). In addition, the expression level of mirNA-1 in the myocardial tissue of the right atrium of group D was lower than that of group B (*P* < 0.05).

(* indicated *P* < 0.05 compared with group A; # meant *P* < 0.05 in contrast to group C; and & represented *P* < 0.05 compared to group B.)

**Figure 9.**
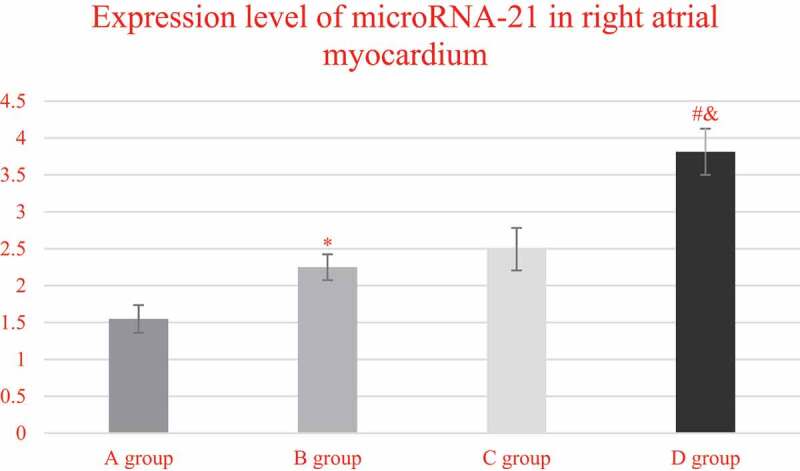
Comparison on the expression levels of miRNA-21 in the right atrium myocardial tissues of the patients from group A, C, and D

Figure 9 demonstrates the comparison on the expression levels of miRNA-21 in the right atrium myocardial tissues of the patients from each group. It indicated that the expression levels of miRNA-21 in the right atrium myocardial tissues of the patients from groups A, B, C, and D were 1.5476 ± 0.1869, 2.2489 ± 0.1756, 2.4928 ± 0.2876, and 3.8127 ± 0.3129, respectively. What is more, the expression level of miRNA-21 in group C was extremely higher than that of group A (*P* < 0.05), and that in group D was obviously higher in contrast to group C (*P* < 0.05). In addition, the expression level of mirNA-21 in the myocardial tissue of the right atrium of group D increased obviously in contrast to the level of group B (*P* < 0.05).

(* indicated *P* < 0.05 compared with group A; # meant *P* < 0.05 in contrast to group C; and & represented *P* < 0.05 compared to group B.)

## 4. Discussion

The prevalence and mortality of AF are both high. With the increase in age, the detection rate of aging AF gradually grows from 0.34% (<60 years old) to 2.73% (≥85 years old) [17]. From that, age is the main risk factor for the onset of aging AF. Therefore, the non-elderly patients with SR, non-elderly patients with AF, elderly patients with SR, and elderly patients with AF were selected to investigate the molecular mechanism of aging atrial remodeling. Atrial electrical remodeling and structural remodeling are the basis of AF, but the exact mechanism driving this remodeling is still not fully clear. Recent studies have shown that miRNA is a short non-coding RNA that regulates gene expression, and may be related to the pathophysiology of AF [18]. The miRNA has great effects on the development of the heart and is correlated with remodeling and fibrosis of the ion channel induced by AF. Shen, et al. (2020) evaluated the miRNA expression profile among the subjects investigated with AF and non-AF and found that miRNA-1-5p up-regulated in the heart tissues, miRNA-21 dysregulated, and five important miRNAs (miR-29b, miR-328, miR-1-5p, miR-21, and miR-223-3p) might be served as potential biomarkers for AF [19]. The expression level of microRNA-1 in group C was remarkably lower than that of group A and group D (*P* < 0.05), while the expression level of miRNA-21 in group C increased markedly in contrast to group A (*P* < 0.05) and dramatically decreased in contrast to group D (*P* > 0.05). It revealed that there were expression differences of miRNA-1 and miRNA-21 in the patients of different ages, SRs, and AFs, which was almost consistent with the results of Ma et al. (2019) [20].

It was discovered that the fibrous tissues among the muscle cells in the right atrium myocardial tissues of the elderly patients were denser and more compact, and many connective tissues separated the muscle bundles, showing the characteristic of excessive accumulation of collagen, which was the most serious in the elderly patients with AF, compared with non-elderly patients. Myocardial fibrosis plays a critical role in the maintenance of AF through the heterogeneity of atrial electrical conduction [21]. During the period of myocardial fibrosis, normal collagen chains in the body are degraded to produce fibrous stromal deposits. Metalloproteinase has a close correlation with the degradation and synthesis of collagen and has marked effects on atrial aging or aging AF. Moreover, these proteolytic enzymes are mainly regulated by TIMPs, and the dynamic balance of MMP-9 and TIMP-1 determines the fibrosis signal strength [22]. The mRNA expression levels of MMP-9, Bcl-2, and Bax in the muscle cells of the right atrium from each group were detected to find that mRNA levels of MMP-9 and Bax in patients with AF were extremely higher than those of patients with sinus rhythm, and mRNA levels of TIMP-1 and Bcl-2 were steeply lower than those of patients with sinus rhythm (*P* < 0.05). It was in accordance with the research results of Diao et al. (2016) [23]. Bcl-2 mainly plays an anti-apoptotic role in myocardial cells, while Bax can promote apoptosis. The middle-aged and elderly patients with AAF in the study had more apoptotic cells, and the expression balance of Bax and Bcl-2 was broken, that is, Bax had the high expression and Bcl-2 had the low expression. Tsoporis et al. detected the levels of miRs 1 and 133A, as well as labeled apoptosis, including TUNEL staining, caspase-3 activation, and mRNA levels of Bcl-2 and Bax. Thus, it was found that miRNA-1 increased and miRNA-133A decreased in the right auricular tissue of patients with AF, which was negatively correlated with the apoptosis of the myocardial tissues of the right atrium [24], which were in line with the conclusion of the study. To sum up, the abnormal expression of miRNA-1 and miRNA-21 may be related to the fibrosis characteristics and the emerging of apoptosis during the process of atrial remodeling.

## 5. Conclusion

Patients with valvular heart disease treated by cardiac surgery were selected as the subjects investigated. The ultrastructure and pathological changes in the myocardial tissue cells of the right atrium were observed by electron microscopy and Masson staining, and the expression levels of miRNA-1 and miRNA-21 in the atrial tissues of the patients were detected. It was found that CVF, apoptotic index AI, and cell ultrastructure showed statistically marked changes with the aggravation of aging and AAF. The expression level of miRNA-l in elderly patients with sinus was greatly lower than that of non-elderly patients with sinus, while the expression level of miRNA-21 in elderly patients with sinus was enormously higher than that of non-elderly patients with sinus (*P* < 0.05). In addition, the expression levels of miRNA-1 and miRNA-21 in the elderly patients with AAF obviously increased in contrast to the elderly patients with sinus (*P* < 0.05). This suggests that microRNA-1 and microRNA-21 are involved in the molecular remodeling of aging atrial fibrillation through the regulation of primers. However, there were some shortcomings in the study, and the specimens were limited to the right auricle. Therefore, several groups of the myocardial tissues from different parts could be added for comparative analysis. Furthermore, it was only to explore the influence mechanism of the miRNA-1 and miRNA-21 expressions on AF. However, its regulatory proteins or related signaling pathways had not been explored, which needed to be further studied and determined. Based on the above results, the study could provide critical evidence for the pathogenesis and clinical treatment of AF.

## Data Availability

The data used to support the findings of this study are included within the article.
